# Supplementary material for: INF2-mediated actin polymerization at ER-organelle contacts regulates organelle size and movement
Source: bioRxiv. 2024 Jul 15:2024.07.06.602365. Originally published 2024 Jul 7. Preprint. [Version 2] doi: 10.1101/2024.07.06.602365 (PMC11245118; doi:10.1101/2024.07.06.602365)
Supplement: 1 [file NIHPP2024.07.06.602365v2-supplement-1.pdf]

bioRxiv preprint doi: https://doi.org/10.1101/2024.07.06.602365; this version posted July 15, 2024. The copyright holder for this preprint (which was not certified by peer review) is the author/funder, who has granted bioRxiv a license to display the preprint in perpetuity. It is made available under aCC-BY 4.0 International license.

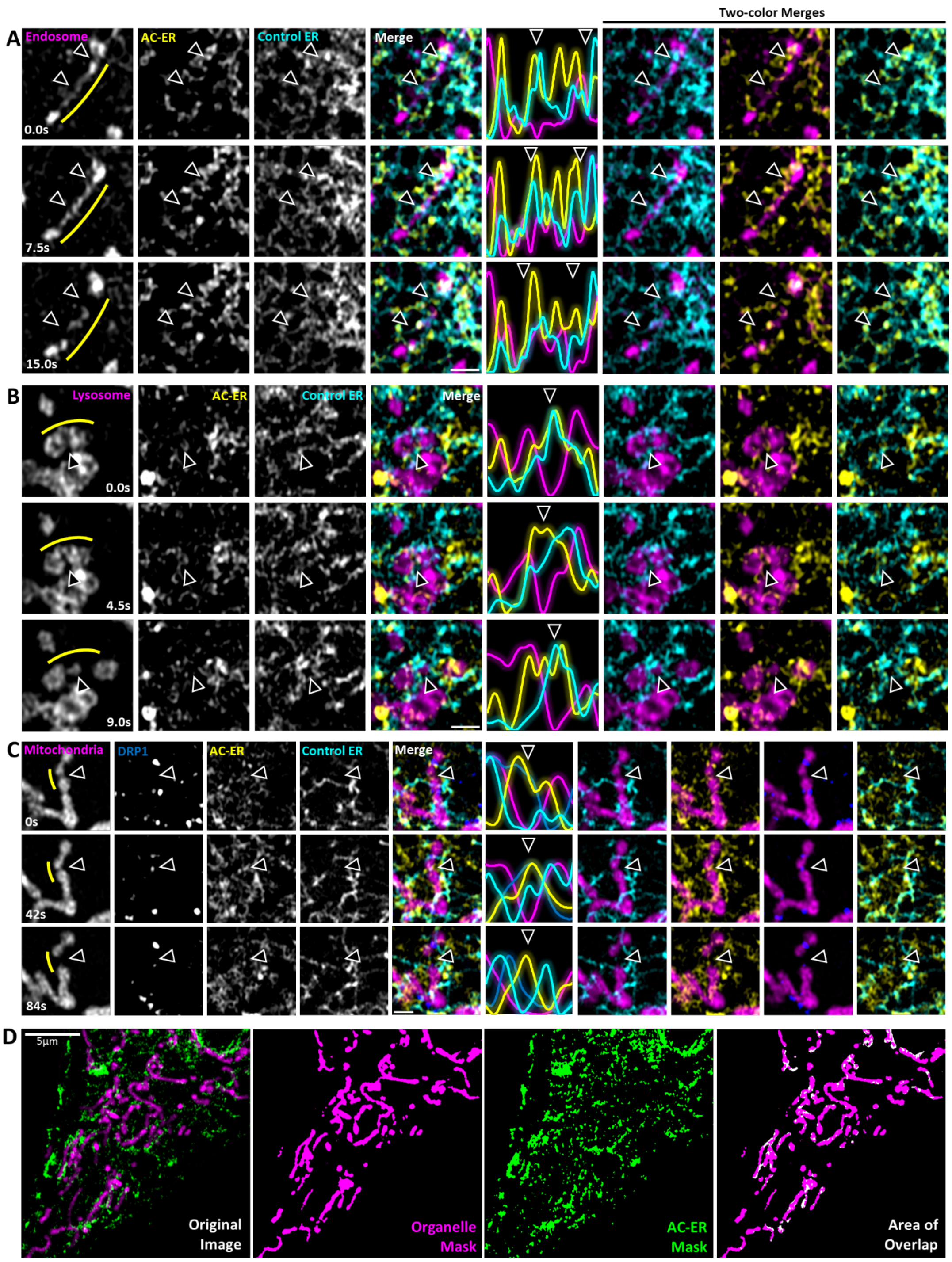

Supp Fig 1: Organelle fission examples displaying all channels and example of masks used to generate "by chance" values. A. The same endosome example from Figure 1A is shown. Like Figure 1, arrowheads denote fission sites, and the yellow line denotes the location where the line scan was drawn. In order from left to right are single channel grayscale images, the merge of all channels (endosomes in magenta, AC-ER in yellow, and ER in cyan), the plot of pixel intensities corresponding to the line scan, and two-channel merges. Scale bar is 1μm. **B.** Same as A but displaying the lysosome example from Figure 1A in the magenta channel. **C.** Similar to A and B but displaying the mitochondria example from Figure 1A in the magenta channel. Additionally, the DRP1 channel is shown in blue. **D.** In the leftmost panel, an example of a typical cell used for analysis of area of overlap between organelles and AC-ER is shown. The organelles used in this example are mitochondria. Mitochondria are labeled in magenta and AC-ER is labeled in green. The appearance of the masks generated by the mitochondrial and AC-ER signals are shown in the center panels. The rightmost panel shows the area of overlap between the mitochondrial mask and AC-ER mask (white), overlaid with the mitochondrial mask (magenta). This area of overlap was used to determine the "by chance" values shown in Figure 1B and C.

bioRxiv preprint doi: https://doi.org/10.1101/2024.07.06.602365; this version posted July 15, 2024. The copyright holder for this preprint (which was not certified by peer review) is the author/funder, who has granted bioRxiv a license to display the preprint in perpetuity. It is made available under aCC-BY 4.0 International license.

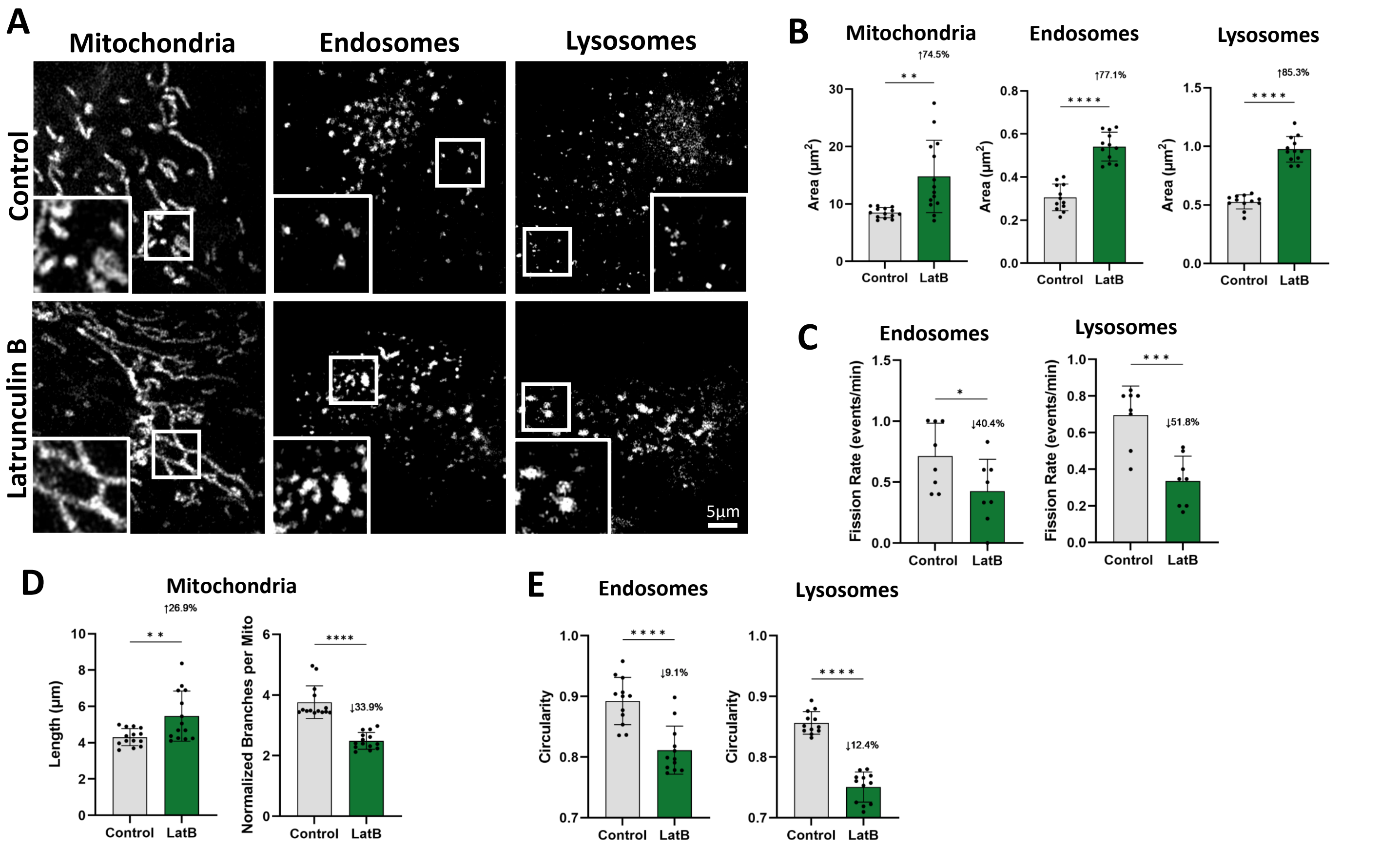

Supp Fig 2: Inhibition of actin polymerization causes organelle enlargement. Organelles in U2OS cells were labeled with Rab5-mCherry (endosomes), LAMP1-mCherry (lysosomes), or MitoTracker Deep Red (mitochondria) and imaged live following addition of 200nM LatB or vehicle control for 60-90 minutes. **A.** Representative images of mitochondria, endosomes, and lysosomes after treatment with vehicle control or LatB are shown. Insets show magnified views of the boxed regions. **B.** Quantification of individual organelle area following treatment with LatB or vehicle control. Mitochondrial morphology was measured using the "Mitochondria Analyzer" Fiji plugin[33]. Endosome and lysosome morphology were measured using a custom Fiji macro which uses a combination of thresholding and marker-controlled watershed to split clustered organelles by using local maxima as seed points, allowing for segmentation of individual organelles. The areas of each individual organelle were measured and the average per cell is shown as black dots on the graphs. Bars show the average across all cells for each condition. Standard deviation is denoted by error bars. Conditions were compared via Welch's t-test. N = 14 cells per condition for mitochondria and 12 per condition for endosomes and lysosomes. **C.** Quantification of manually scored fission rates in endosomes and lysosomes following LatB or vehicle control treatment. N = 8 cells per condition. **D.** Quantification of mitochondrial length and number of mitochondrial branches following LatB or vehicle control treatment. N = 14 cells per condition. **E.** Quantification of endosome and lysosome circularity following LatB or vehicle control treatment. N = 12 cells per condition. For all graphs, the magnitude of the change compared to control is shown. An up arrow indicates an increase and a down arrow indicates a decrease. For statistical comparisons **** indicates p-value≤0.0001, *** indicates p-values≤0.001, ** indicates p-value≤0.01, and * indicates p-value≤0.05. All conditions were compared via Welch's t-test. All experiments were performed with N=3 biological replicates.

bioRxiv preprint doi: https://doi.org/10.1101/2024.07.06.602365; this version posted July 15, 2024. The copyright holder for this preprint (which was not certified by peer review) is the author/funder, who has granted bioRxiv a license to display the preprint in perpetuity. It is made available under aCC-BY 4.0 International license.

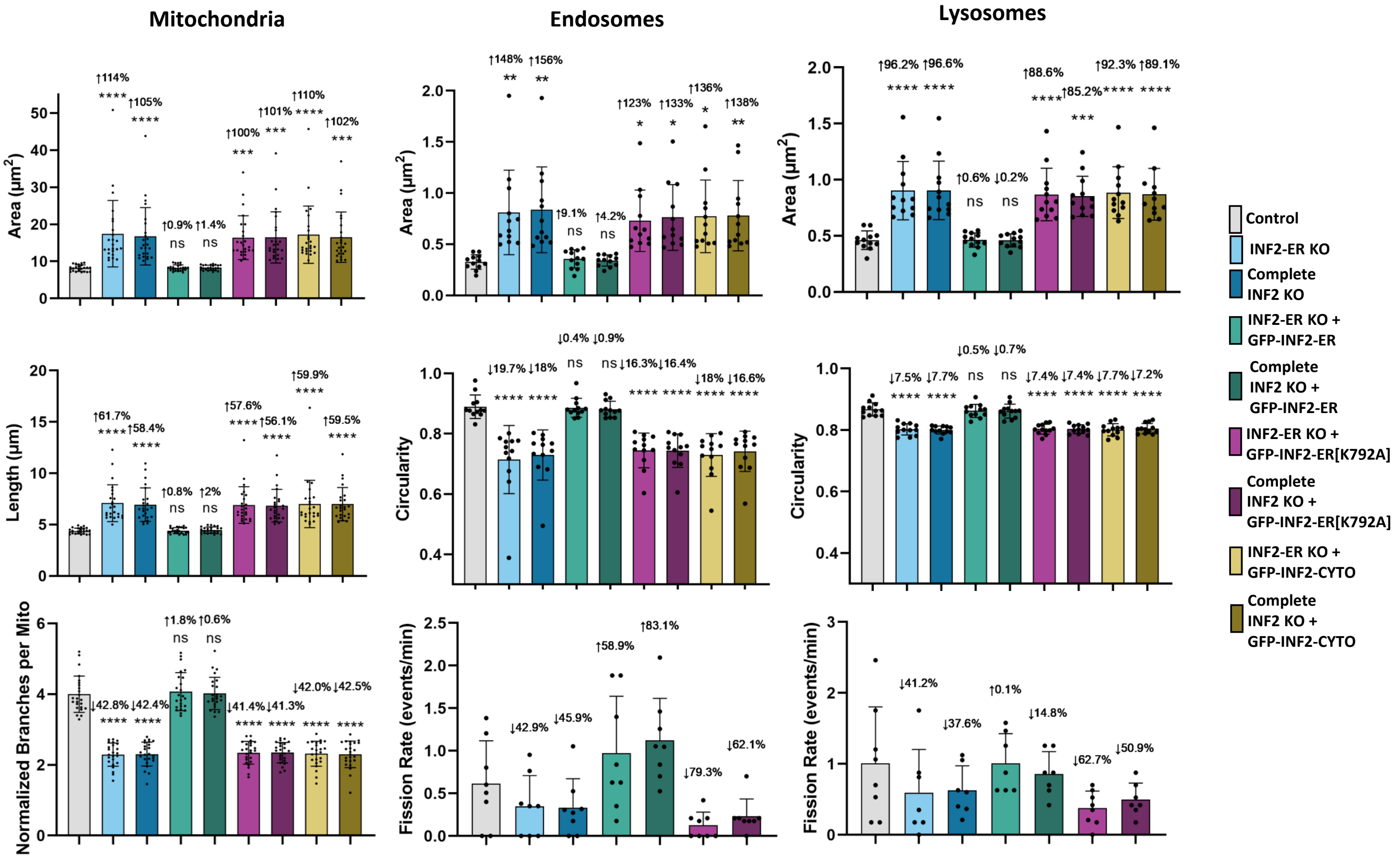

Supp Fig 3: Further quantification of the effect of INF2 knock-out on organelle morphology. Additional quantification results related to the experiments described in Figure 2 are shown. The top row of graphs shows the same individual organelle area quantification results from Figure 2 with the addition of results from experiments carried out with U2OS cells where all isoforms of INF2 have been knocked-out ("Complete INF2 KO"). In addition to area, individual mitochondrial length, the number of branches per mitochondria, and circularity of endosomes and lysosomes are also shown. Endosome and lysosome fission rates are shown in the bottom right graphs. N = 8 cells per condition for fission rate results. For all other results, N = 12 cells per condition for endosomes and lysosomes and 24 cells per condition for mitochondria. For all graphs, black dots show the average per cell and bars indicate the average per condition. Error bars show standard deviation. The magnitude of the change and p-value compared to control is shown. An up arrow indicates an increase and a down arrow indicates a decrease. For statistical comparisons **** indicates p-value≤0.0001, *** indicates p-values≤0.001, ** indicates p-value≤0.01, * indicates p-value≤0.05, and ns indicates p-value>0.05. Conditions were compared via ordinary one-way ANOVA. All experiments were performed with N=3 biological replicates.

bioRxiv preprint doi: https://doi.org/10.1101/2024.07.06.602365; this version posted July 15, 2024. The copyright holder for this preprint (which was not certified by peer review) is the author/funder, who has granted bioRxiv a license to display the preprint in perpetuity. It is made available under aCC-BY 4.0 International license.

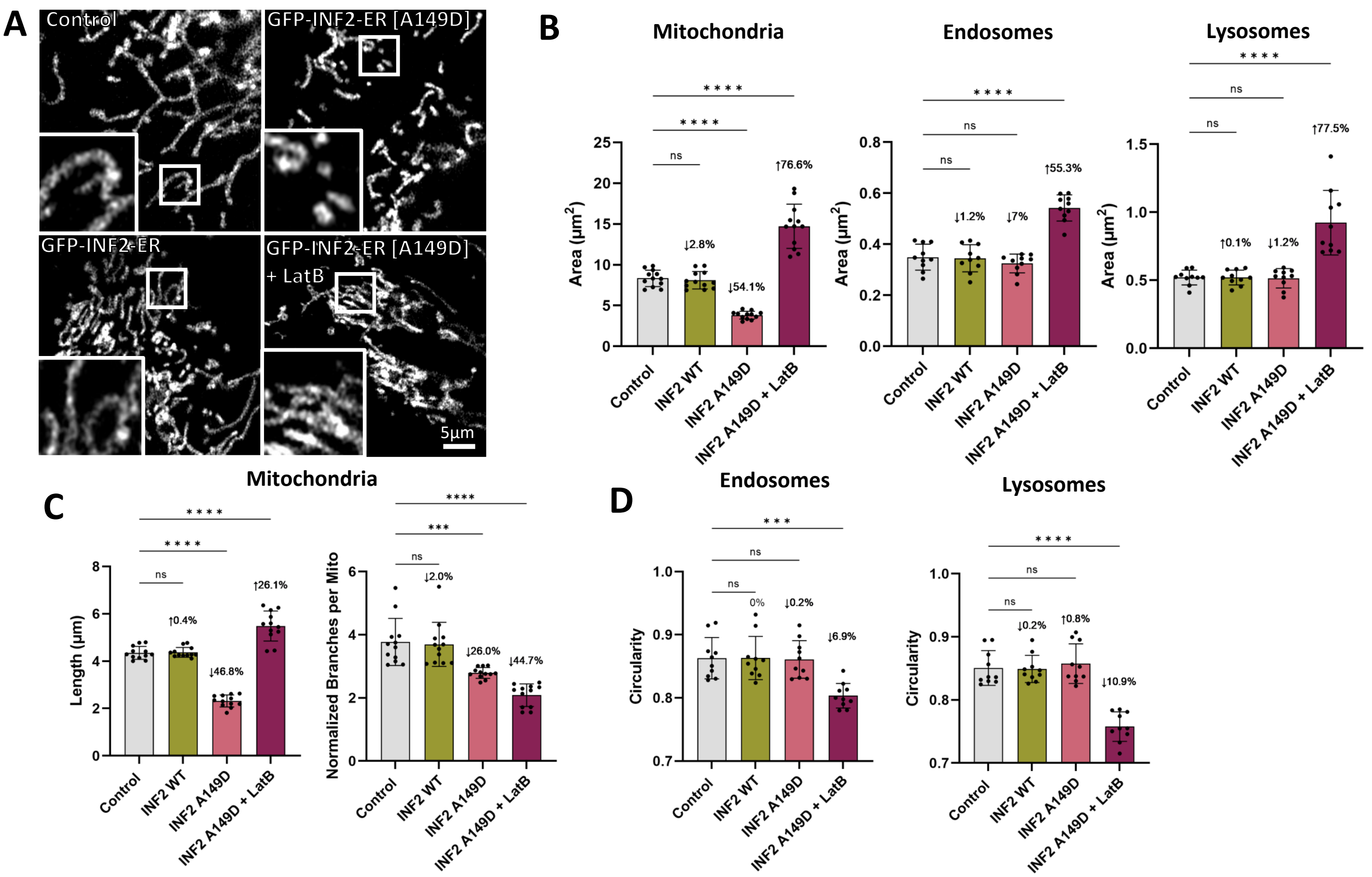

Supp Fig 4: Expression of dominant active INF2 causes mitochondrial fragmentation but does not alter endosome or lysosome shape. Wild-type U2OS cells were imaged live following co-transfection with organelle labels and various INF2-ER constructs. **A.** Representative images of mitochondria labeled with MitoTracker Deep Red in wild-type U2OS cells expressing GFP (control), wild-type INF2-ER GFP, or INF2-ER[A149D] GFP are shown. A representative image of mitochondria from the INF2-ER[A149D] GFP condition following treatment with 1μM LatB for 15-30 minutes is also shown. **B.** Quantification of individual organelle area for each condition and organelle. **C.** Quantification of mitochondrial length and branching for each condition. **D.** Quantification of endosome and lysosome circularity for all conditions. For all graphs, black dots show the average per cell and bars indicate the average per condition. Error bars show standard deviation. The magnitude of the change and p-value compared to control is shown. An up arrow indicates an increase and a down arrow indicates a decrease. For statistical comparisons **** indicates p-value≤0.0001, *** indicates p-values≤0.001, ** indicates p-value≤0.01, * indicates p-value≤0.05, and ns indicates p-value>0.05. Conditions were compared via ordinary one-way ANOVA. N = 10 cells per condition for endosomes and lysosomes and 12 cells per condition for mitochondria. All experiments were performed with N=3 biological replicates.

bioRxiv preprint doi: https://doi.org/10.1101/2024.07.06.602365; this version posted July 15, 2024. The copyright holder for this preprint (which was not certified by peer review) is the author/funder, who has granted bioRxiv a license to display the preprint in perpetuity. It is made available under aCC-BY 4.0 International license.

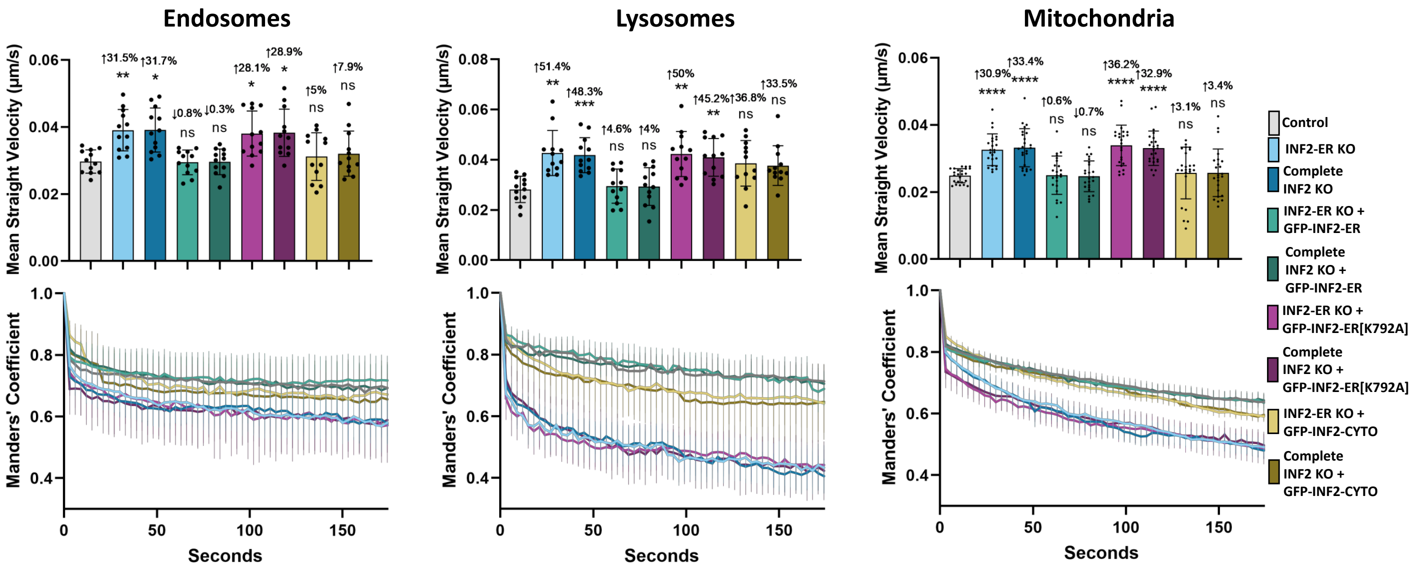

P-values for each curve compared to Control curve			
INF2-ER KO	$1.5x10^{-8}$	Complete INF2 KO	$<1x10^{-15}$
INF2-ER KO + GFP-INF2-ER	1	Complete INF2 KO + GFP-INF2-ER	1
INF2-ER KO + GFP-INF2-ER[K792A]	$4.4x10^{-2}$	Complete INF2 KO + GFP-INF2-ER[K792A]	$6.0x10^{-4}$
INF2-ER KO + GFP-INF2-CYTO	1	Complete INF2 KO + GFP-INF2-CYTO	1

P-values for each curve compared to Control curve			
INF2-ER KO	$<1x10^{-15}$	Complete INF2 KO	$<1x10^{-15}$
INF2-ER KO + GFP-INF2-ER	1	Complete INF2 KO + GFP-INF2-ER	1
INF2-ER KO + GFP-INF2-ER[K792A]	$<1x10^{-15}$	Complete INF2 KO + GFP-INF2-ER[K792A]	$<1x10^{-15}$
INF2-ER KO + GFPINF2-CYTO	$4.6x10^{-1}$	Complete INF2 KO + GFP-INF2-CYTO	$7.2x10^{-1}$

P-values for each curve compared to Control curve			
INF2-ER KO	$<1x10^{-15}$	Complete INF2 KO	$<1x10^{-15}$
INF2-ER KO + GFP-INF2-ER	1	Complete INF2 KO + GFP-INF2-ER	1
INF2-ER KO + GFP-INF2-ER[K792A]	$<1x10^{-15}$	Complete INF2 KO + GFP-INF2-ER[K792A]	$<1x10^{-15}$
INF2-ER KO + GFP-INF2-CYTO	$1.3x10^{-6}$	Complete INF2 KO + GFP-INF2-CYTO	$9.7x10^{-3}$

Supp Fig 5: Further quantification of the effect of INF2 KO on organelle mobility. Additional quantification results related to the experiments described in Figure 3 are shown. The top row of graphs shows the same mean straight velocity values from Figure 3 with the addition of values from experiments carried out in Complete INF2 KO cells. Black dots show the average per cell and bars indicate the average per condition. Error bars show standard deviation. The magnitude of the change and p-value compared to control is shown. An up arrow indicates an increase and a down arrow indicates a decrease. For statistical comparisons **** indicates p-value≤0.0001, *** indicates p-values≤0.001, ** indicates p-value≤0.01, * indicates p-value≤0.05, and ns indicates p-value>0.05. Conditions were compared via ordinary one-way ANOVA. The second row of graphs shows the autocorrelation values of the organelle channel over time for each condition calculated using a custom Fiji macro described in Methods. The thick lines mark the average correlation value over time and the thin vertical lines show the standard error. Tables below the graphs show the p-value results of comparing each curve to the control curve. Curves were compared using a modified Chi-squared method[30]. For all graphs, N = 12 cells per condition for endosomes and lysosomes and 24 cells per condition for mitochondria. All experiments were performed with N=3 biological replicates.

bioRxiv preprint doi: https://doi.org/10.1101/2024.07.06.602365; this version posted July 15, 2024. The copyright holder for this preprint (which was not certified by peer review) is the author/funder, who has granted bioRxiv a license to display the preprint in perpetuity. It is made available under aCC-BY 4.0 International license.

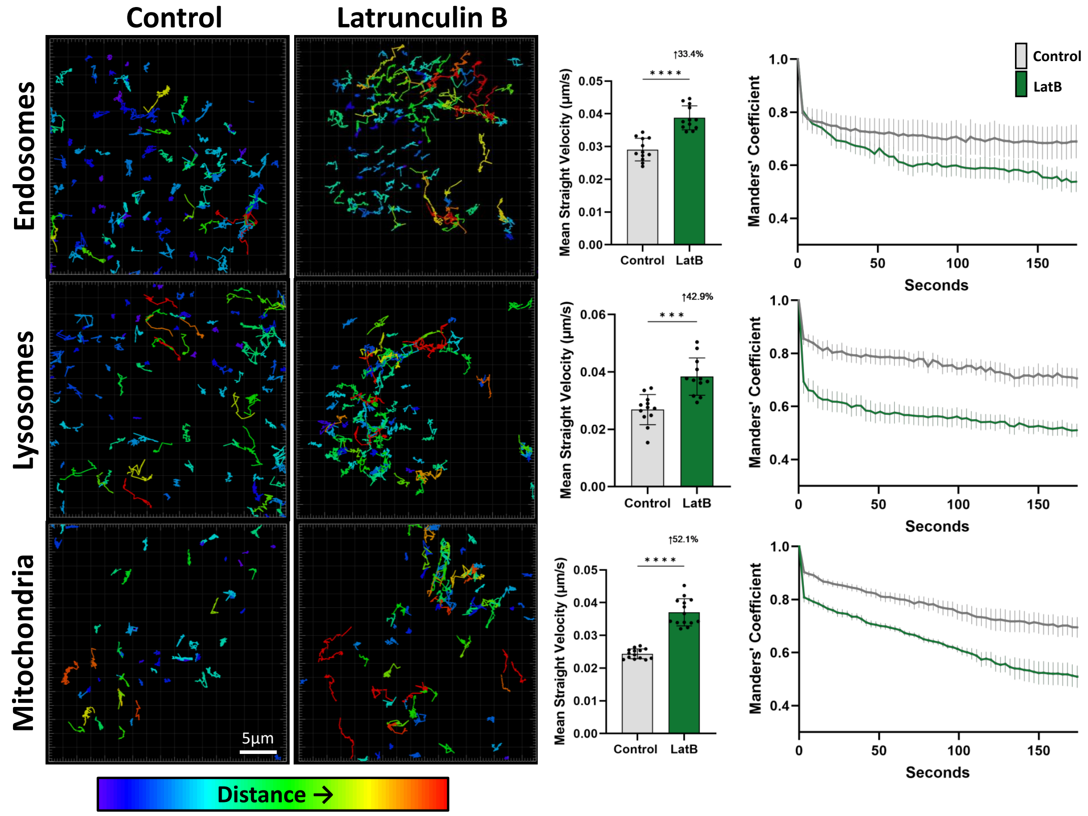

P-values for LatB curve compared to Control curve	
Endosomes	$2.9x10^{-11}$
Lysosomes	$<1x10^{-15}$
Mitochondria	$<1x10^{-15}$

Supp Fig 6: Inhibition of actin polymerization increases organelle mobility. Wild-type U2OS cells were labeled with Rab5-mCherry (endosomes), LAMP1-mCherry (lysosomes), or MitoTracker Deep Red (mitochondria), treated with 200nM LatB or vehicle control for 60-90 minutes, then imaged over time for an additional 5 minutes. Organelle movement was measured using both Imaris tracking and our autocorrelation method. Panels display tracks of individual organelle trajectories color-coded based on distance traveled as described in Figure 3. Graphs of organelle tracking results are also displayed. Black dots show the average per cell and bars indicate the average per condition. Error bars show standard deviation. The magnitude of the change and p-value compared to control is shown. An up arrow indicates an increase and a down arrow indicates a decrease. For statistical comparisons **** indicates p-value≤0.0001, *** indicates p-values≤0.001, ** indicates p-value≤0.01, * indicates p-value≤0.05, and ns indicates p-value>0.05. Conditions were compared via Welch's t-test. Graphs of the autocorrelation values over time are also shown. The thick lines mark the average correlation value over time and the thin vertical lines show the standard error. The table displays the p-values resulting from comparing the control and LatB curves. For all graphs, N = 12 cells per condition for endosomes and lysosomes and 14 cells per condition for mitochondria. All experiments were performed with N=3 biological replicates.

bioRxiv preprint doi: https://doi.org/10.1101/2024.07.06.602365; this version posted July 15, 2024. The copyright holder for this preprint (which was not certified by peer review) is the author/funder, who has granted bioRxiv a license to display the preprint in perpetuity. It is made available under aCC-BY 4.0 International license.

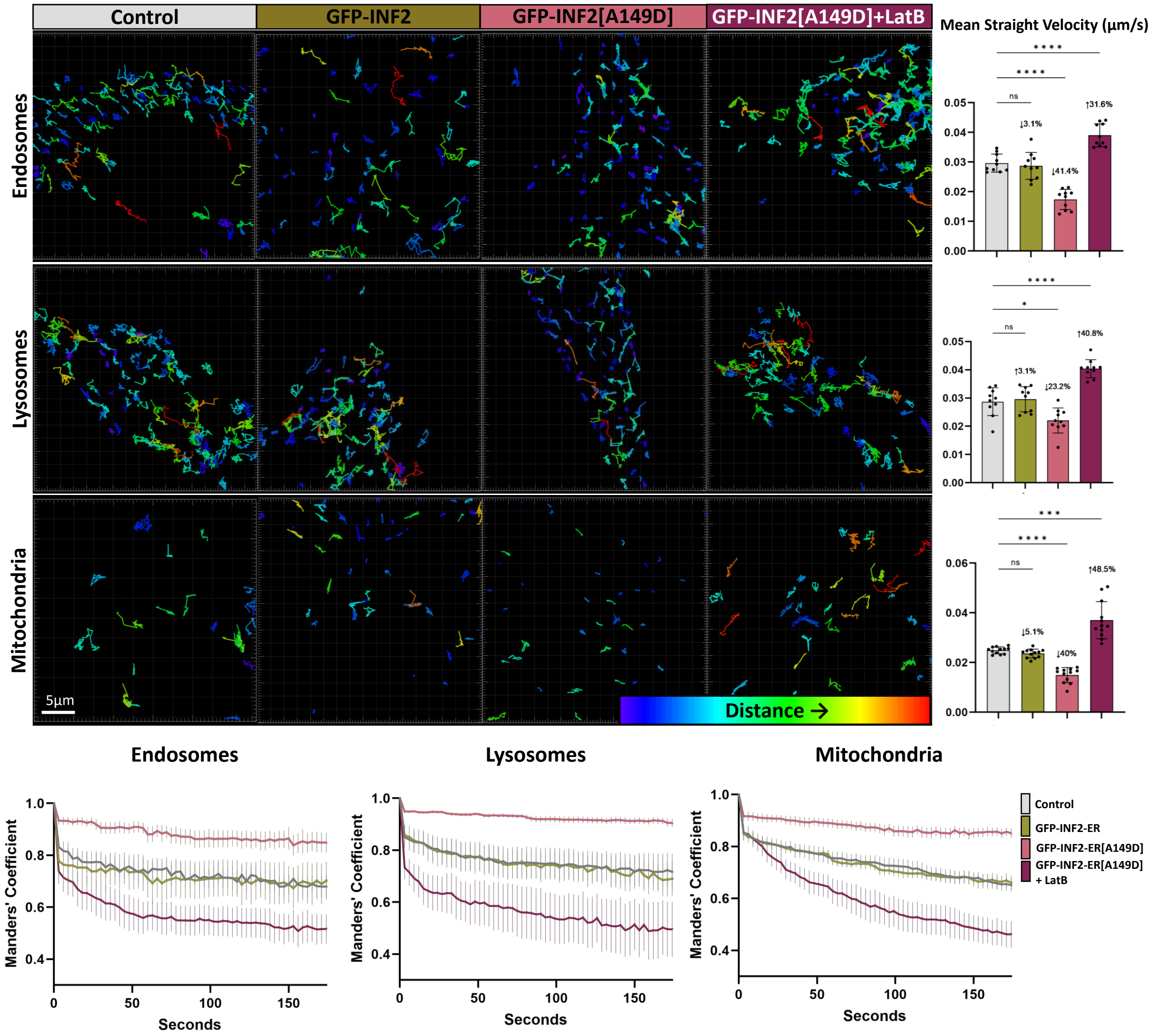

P-values for each curve compared to Control curve	
GFP-INF2-ER	1
GFP-INF2-ER[A149D]	$<1x10^{-15}$
GFP-INF2-ER[A149D]+LatB	$<1x10^{-15}$

P-values for each curve compared to Control curve	
GFP-INF2-ER	1
GFP-INF2-ER[A149D]	$<1x10^{-15}$
GFP-INF2-ER[A149D]+LatB	$<1x10^{-15}$

P-values for each curve compared to Control curve	
GFP-INF2-ER	1
GFP-INF2-ER[A149D]	$<1x10^{-15}$
GFP-INF2-ER[A149D]+LatB	$<1x10^{-15}$

Supp Fig 7: Expression of dominant active INF2 reduces organelle mobility. In wild-type U2OS cells organelles were labeled using Rab5-mCherry (endosomes), LAMP1-mCherry (lysosomes), or MitoTracker Deep Red (mitochondria) and co-transfected with GFP (control), wild-type INF2-ER GFP, or INF2-ER[A149D] GFP. The INF2-ER[A149D] GFP condition was also compared with and without 15-30 minute treatment with 1µM LatB. Tracks displaying individual organelle trajectories color-coded by distance traveled are shown for representative cells from each condition. Quantification of these results via organelle tracking in Imaris is shown in the bar graphs. For all bar graphs, black dots show the average per cell and bars indicate the average per condition. Error bars show standard deviation. The magnitude of the change and p-value compared to control are shown. An up arrow indicates an increase and a down arrow indicates a decrease. For statistical comparisons, **** indicates p-value≤0.0001, *** indicates p-values≤0.001, ** indicates p-value≤0.01, * indicates p-value≤0.05, and ns indicates p-value>0.05. Conditions were compared via ordinary one-way ANOVA. In addition to object tracking in Imaris, organelle movement was also quantified using our autocorrelation method, displayed in the line graphs. The thick lines mark the average correlation value over time and the thin vertical lines show the standard error. The tables display the p-values resulting from comparing each curve with the control curve. N = 10 cells per condition for endosomes and lysosomes and 12 cells per condition for mitochondria. All experiments were performed with N=3 biological replicates.
